# The Many Pathways to Personal Growth: Contemporary Perspectives and Future Directions

**DOI:** 10.3390/bs16091692

**Published:** 2026-09-20

**Authors:** Madoka Kumashiro

**Affiliations:** Department of Psychology and Neuroscience, Goldsmiths, University of London, London SE14 6NW, UK; madoka.kumashiro@gold.ac.uk

**Keywords:** personal growth, well-being, eudaimonia, post-traumatic growth, motivation, close relationships, social support, artificial intelligence, individual differences, attachment

## Abstract

Although philosophers and psychologists have pondered for centuries on what it means to pursue personal development and to live a good life, psychological research on personal growth remains broad and diverse. This Editorial introduces a Special Issue comprising 25 contributions to *Experiences and Well-Being in Personal Growth*. These diverse perspectives conceptualize growth as an aspect of eudaimonic well-being, an intentional process of self-change, a response to adversity, or a process facilitated by relationships and broader contexts. Growth may emerge through coping with adversity or through engagement with life opportunities, and its likelihood and consequences depend on individual characteristics, close relationships, and wider social and cultural environments. The Editorial synthesizes recent developments in the field, with special attention paid to the central role of interpersonal relationships, highlighting contributions from this Special Issue to the broader discussion surrounding personal growth. Future research directions are identified, including the need to prioritize greater conceptual and measurement precision, stronger longitudinal and causal evidence, closer attention to individual and contextual variation, and consideration of how the growing use of conversational AI for support and guidance may reshape how personal growth is pursued, experienced, and understood.

## 1. Introduction

How do people experience personal growth? What does it mean to continue to develop as a person and to live a meaningful life? And how, if at all, does such development contribute to living well? Questions concerning personal growth have long occupied scholarly thought and have become increasingly prominent in popular culture. Although contemporary interest is evident in the proliferation of books, digital apps, and other resources devoted to self-improvement and well-being, philosophical and religious traditions and generations of scholars have addressed these questions concerning personal development and the good life for centuries. For example, as Ryff (2026; Contribution 18) notes in her review of eudaimonic personal growth in this Special Issue, Aristotle pondered how humans should live, whether living well entails being happy, what happiness itself means, and whether a good life instead requires the cultivation of virtuous character. These questions helped lay the groundwork for the contemporary distinction between hedonic approaches to well-being, which emphasize experiences such as happiness, pleasure, and positive affect, and eudaimonic approaches, which place greater emphasis on optimal functioning, meaning, realization of potential, and becoming the kind of person one is capable of being.

As Ryff (2026; Contribution 18) further notes, influential psychological theorists of the mid-20th century, including Maslow, Rogers, Jung, von Franz, and Allport, developed accounts of human development and optimal functioning, using a variety of terms such as self-actualization, the fully functioning person, maturity, and individuation. Ryff’s multidimensional model of psychological well-being (Ryff, 1985, 1989) was particularly influential in translating these diverse theoretical traditions into an empirically measurable framework, conceptualizing personal growth as one of the six components of well-being. Yet, this positioning of personal growth within well-being is not universal. Other theoretical traditions instead emphasize motivational or contextual conditions that foster growth, intentional efforts to change and develop, or positive changes that emerge following significant life experiences. Thus, despite its intuitive familiarity, personal growth has no single agreed psychological definition, nor is its relationship with well-being settled.

This Editorial considers some of the recent research developments on personal growth^1^, highlighting contributions from the Special Issue on *Experiences and Well-Being in Personal Growth* to examine how growth is conceptualized, experienced, and supported across diverse life circumstances. Across these diverse perspectives, one recurring theme is that personal growth is not solely an intrapersonal process: relationships and wider social and ecological contexts can facilitate, constrain, or shape how growth occurs. The Editorial concludes by identifying priorities for future research, paying particular attention to cultural, societal, and technological changes that may reshape how personal growth is pursued and experienced.

## 2. Major Contemporary Perspectives on Personal Growth

Even as research and public interest in personal growth related topics increases rapidly, there seems to be no consensus on how to define personal growth and what constitutes personal growth. This ambiguity is evident even in general reference sources: despite widespread use of the term, personal growth is not consistently defined as a distinct concept, with one dictionary providing a standalone but broad definition of “development as an individual” (Collins English Dictionary, n.d.). A similar lack of conceptual consensus is evident within psychology. Across psychology, personal growth and closely related constructs are conceptualized at different levels: as an aspect of well-being, a developmental process or outcome, an intentional effort toward self-change, a belief about the possibility of change, positive change following adversity, or something facilitated by relationships and broader contexts. Although these traditions share a broad concern with continued human development, they have developed largely along separate theoretical tracks, emphasizing eudaimonic well-being (e.g., Ryff, 1989), conditions that facilitate optimal development and functioning (e.g., Deci & Ryan, 2000), intentional agency (e.g., personal growth initiative; Robitschek et al., 2012), belief structures (e.g., growth mindset; Dweck, 2006), adversity-driven change (e.g., posttraumatic growth; Tedeschi & Calhoun, 2004), and an emphasis on relationships as a driver of personal growth and flourishing (e.g., Feeney & Collins, 2015).

One major source of conceptual ambiguity concerns whether personal growth should itself be regarded as a component of well-being or as a process or condition that contributes to well-being. Within Ryff’s (1989) influential model of well-being, as reviewed in this Special Issue (Ryff, 2026; Contribution 18), personal growth primarily refers to the extent to which individuals perceive themselves as continuing to develop and realize their potential, with personal growth constituting one of the six distinct dimensions of well-being, alongside autonomy, environmental mastery, purpose in life, self-acceptance, and positive relations with others. Thus, within this framework, personal growth is itself part of what it means to function well.

Alternatively, self-determination theory (SDT; Deci & Ryan, 2000; Ryan & Deci, 2001), a major comprehensive theory of human motivation and development, positions these constructs differently. Rather than treating autonomy, competence, relatedness, and personal growth as parallel dimensions of well-being, SDT identifies autonomy, competence, and relatedness as basic psychological needs, the satisfaction of which supports autonomous motivation, integration, healthy development, and well-being. These different formulations illustrate a broader unresolved question: whether personal growth is best conceptualized as part of well-being itself, as an outcome of conditions that foster optimal functioning, or as a developmental process that contributes to well-being.

In contrast, instead of treating personal growth as a core feature of well-being, personal growth initiative (PGI) focuses on the process of intentional growth, emphasizing individuals’ active and intentional involvement in changing and developing themselves, incorporating cognitive and behavioral processes such as readiness for change, planning abilities, resource use, and intentional action (Robitschek et al., 2012). PGI does not measure whether people have grown so much as their intentional engagement in processes intended to produce personal change. Consistent with this distinction, Cui et al. (2026; Contribution 6) examined PGI as a pathway linking features of school climate with depressive symptoms and subjective well-being, conceptually positioning growth initiative as a process rather than an indicator of well-being.

Similarly, the growth mindset draws on implicit theories of intelligence and ability (e.g., Dweck & Leggett, 1988; Dweck, 2006) to emphasize the importance of developing a belief system that a person can continue to develop through effort, rather than believing that one’s abilities are inherently fixed and cannot be changed. Research has proliferated on how to help individuals adopt a growth mindset, but meta-analyses and reviews on growth mindset interventions show mixed findings (Burnette et al., 2023; Dweck & Yeager, 2019). Combining the belief systems and process-oriented approaches, Bobinet et al. (2025; Contribution 4) introduce and develop a new Iterative Mindset Inventory, which focuses more specifically on how people respond when self-improvement efforts fail, through a process of iteration, practice and assessment.

Moreover, other psychological theories focus on specific conditions under which personal growth may occur, especially following adverse experiences. For example, post-traumatic growth theory (PTG) emerged from a separate clinical domain, focusing on positive changes that may arise from traumatic or highly challenging experiences rather than development arising from ordinary opportunities (Tedeschi & Calhoun, 2004; for a review, see Tedeschi et al., 2025; Contribution 22). PTG therefore specifies both a putative form of growth and a class of circumstances under which such growth is experienced, whereas Ryff’s (1989) personal growth construct and PGI (Robitschek et al., 2012) do not require prior adversity. Rather than treating adversity itself as growth-producing, PTG theory emphasizes the cognitive, emotional, and relational work through which disrupted assumptions about the self and world may be reconstructed. PTG has been distinguished from other psychological constructs related to adversity. For example, Taku and Efthemiou (2025; Contribution 20) provide further support for research distinguishing PTG from similar constructs like resilience, as the two show different patterns of associations with optimism and pessimism. The next section will describe such adversity-related growth processes in more detail, highlighting the many contributions in this Special Issue that touch on this topic.

Finally, while many theories of personal growth focus on within-person processes (e.g., strengthening autonomous motivation, personal growth initiative, iterative or growth mindset, competence, self-regulation, etc.), an increasingly prominent complementary perspective asks how growth is enabled, constrained, or co-constructed through relationships. While some of the major theories discussed already incorporate relationships as an integral aspect of well-being (e.g., Ryff, 1989), a fundamental human need (e.g., Deci & Ryan, 2000; Ryan & Deci, 2001), or a facilitator of PTG (e.g., Tedeschi & Calhoun, 2004; Tedeschi et al., 2025; Contribution 22), Feeney and Collins (2015) proposed a comprehensive interpersonal model of thriving, where relationships and social connections are central for personal growth. Complementary theories in relationship research identify several mechanisms through which close others may support personal development, such as attachment theory’s secure base (e.g., Mikulincer & Shaver, 2016; Feeney & Collins, 2015), self-expansion or self-change through others (e.g., Aron & Aron, 1986; Mattingly et al., 2014; Aron & Tomlinson, 2026; Contribution 1), and partner affirmation of the ideal self (e.g., Drigotas et al., 1999; Rusbult et al., 2009). The importance of the broader social ecology of personal growth is considered later.

In particular, the interpersonal model of thriving (Feeney & Collins, 2015) offers a useful framework for integrating many themes that arise from research on personal growth-related constructs. Rather than viewing social support primarily as a resource that buffers individuals against distress, their model proposes that responsive relationships facilitate thriving in two distinct life contexts: by providing a Source of Strength (SOS) when individuals encounter adversity and by acting as a Relational Catalyst (RC) that encourages exploration, goal pursuit, and opportunities for growth in the absence of adversity. Importantly, this model does not imply that one romantic partner is necessary for development; individuals are most likely to thrive when embedded within networks of responsive relationships that may include partners, family members, friends, siblings, mentors, and work colleagues.

Collectively, these perspectives illustrate that personal growth does not currently refer to a single psychological construct or level of analysis. It may describe an aspect of well-being, intentional engagement in self-change, beliefs about the possibility of change, positive change following adversity, or development facilitated through interpersonal and broader contextual processes. The challenge for future research may therefore be less to impose a single definition than to clarify how these related constructs differ, overlap, and operate together. One particularly useful point of convergence, especially for the purpose of synthesizing the 25 articles from the Special Issue, concerns the social contexts in which growth occurs. The next sections use Feeney and Collins’s (2015) distinction to examine contributions to the Special Issue concerning growth in the face of adversity and growth through engagement with life’s opportunities.

## 3. Growth in the Face of Adversity

Many theories of personal growth and human flourishing (e.g., see Feeney & Collins, 2015 for review) recognize that development can occur in the context of stress, adversity, and major life challenges, while also emphasizing that positive changes are not inevitable. Rather, whether adverse experiences contribute to growth depends on how individuals respond to and make sense of their experiences, often with the help of close others. This starts in early childhood, with theories like attachment theory (e.g., Bowlby, 1988) showing the importance of having responsive caregivers who can provide a secure base from which children can explore unfamiliar and potentially risky environments while remaining confident that support is available when needed. The process continues as an adult, with attachment figures providing a similar secure base for exploration (Mikulincer & Shaver, 2016).

In Feeney and Collins’s (2015) model, close others can provide “Source of Strength” (SOS) support during adverse circumstances, which extends beyond providing safe haven functions described in attachment theory (Bowlby, 1988). Responsive others can help individuals regulate distress, recognize personal resources, reframe difficult circumstances, and engage constructively with the challenges they face. Similarly, PTG theory, which emerged separately from a clinical context, also emphasizes the importance of such supportive others, or expert companions, to aid in the growth process after particularly traumatic circumstances (see Tedeschi et al., 2025; Contribution 22). Interestingly, Greene et al. (2025; Contribution 10) extend this relational perspective by showing that peer-support specialists who were indirectly exposed to others’ trauma may themselves report vicarious PTG, such as challenges to core beliefs and cognitive/emotional changes that appear to parallel the PTG process.

Importantly, social relationships are not only resources for coping with adversity; interpersonal and social contexts can themselves generate or intensify the challenges through which growth is subsequently negotiated. Parenthood is one such context, from women undergoing stressful medically assisted reproduction (Mijalevich-Soker et al., 2025; Contribution 14) to finding gender differences during a demanding period of transition to first-time parenthood (Eliakim et al., 2026; Contribution 7). Moreover, other people may intentionally cause adverse contexts, such as in the case of experienced discrimination, which is associated with the perception of both PTG and depreciation (Boals et al., 2026; Contribution 3).

At the same time, not every socially oriented coping style is associated with PTG after traumatic experiences. Kruger (2026; Contribution 12) presents findings that self-enhancing humor, an intrapersonal coping style involving the maintenance of perspective and positive affect in the face of difficulty, was positively associated with all five PTG domains, whereas affiliative humor, a more socially oriented humor style, was not independently associated with any PTG dimension. Combined with theories of secure-base support and the role of expert companions in PTG (e.g., Mikulincer & Shaver, 2016; Tedeschi et al., 2025; Contribution 22), these findings suggest that relationships may facilitate growth not simply by providing social connection or maintaining relationship quality, but when they help individuals engage constructively with adversity: for example, by restoring sufficient security to confront difficult experiences, encouraging reflection and meaning making, working through or reconstructing disrupted assumptions, and helping individuals recognize new perspectives or personal resources.

Thus, it is important to differentiate adverse experiences from growth that could follow. Challenging circumstances can disrupt assumptions, identities, goals, or relationships and thereby create conditions in which change becomes possible, but the direction and extent of such change appear to depend on how individuals process those experiences and on the resources available to them. Importantly, the interpersonal contribution to growth may depend less on the mere presence of supportive relationships than on the functions those relationships provide, i.e., the type of SOS support (Feeney & Collins, 2015) that enables individuals to engage with difficult experiences, reconsider disrupted assumptions, recognize personal resources, and construct new meaning may be more consequential for growth than social connection alone.

## 4. Growth Through Engagement with Life’s Opportunities

Growth does not necessarily emerge from overcoming adversity. Feeney and Collins’s (2015) interpersonal model of thriving identifies a complementary pathway in which individuals develop through engagement with life opportunities, including exploration, goal pursuit, and novel or challenging experiences. In these circumstances, close others can serve as “Relational Catalysts” (RCs), encouraging individuals to pursue potentially growth-enhancing opportunities and providing the support needed to engage with them effectively. Thus, relationships may contribute to personal growth not only reactively, by helping individuals cope with adversity, but also proactively, by enabling individuals to expand their capacities and move toward personally meaningful goals.

Some contributions from this Special Issue highlight and extend such examples of relationship-facilitated growth. For example, the self-expansion model describes a motivation to increase one’s resources, perspectives, identities, and capabilities, including through close relationships (Aron & Aron, 1986), and Aron and Tomlinson’s (2026; Contribution 1) review shared self-expanding activities as one route from relationship functioning to personal well-being. Relational support may also facilitate growth through assistance with personally important goals, although the effectiveness of such support depends on what close others actually do. Pauw et al. (2026; Contribution 15) examine how different interpersonal emotion-regulation strategies during goal pursuit may serve hedonic and instrumental goals differently.

Similarly, the Michelangelo phenomenon, another interpersonal model of personal growth, describes a behavioral confirmation process, whereby affirmation of the ideal self from close others facilitates movement toward one’s own ideal self and enhances personal and relational well-being (Drigotas et al., 1999; Rusbult et al., 2009). Gu et al. (2026; Contribution 11) present findings that attachment security was associated with greater partner affirmation. These findings further illustrate that the presence of a close relationship alone may not be sufficient for personal growth; individual and relational characteristics can shape whether growth-supportive interpersonal processes occur.

The distinction between growth through adversity and growth through life opportunities should not, however, be regarded as absolute. Directly applying the framework of Feeney and Collins (2015), Baron et al. (2026; Contribution 2) show that for sexually and gender-diverse young adults, the presence of identity-affirming close others may contribute both to coping with stigma-related stressors through SOS support and to personal growth and thriving through RC support. These findings illustrate how individuals confronting stigma-related adversity may simultaneously receive support for coping with stressors and support for pursuing opportunities for growth. Other research also supports dual pathways for growth related processes: for example, close partners can help individuals become more secure in their attachment over time (Arriaga et al., 2018), through targeted strategies aimed at reducing attachment anxiety and avoidance during conflictual moments and increasing security in calmer moments. This process may ultimately aid in personal growth as attachment security has been robustly associated with many indicators of personal growth and flourishing (e.g., Mikulincer & Shaver, 2016).

Some contributions to this Special Issue further raise the possibility that growth-facilitating functions traditionally examined within human relationships may extend to non-human relationships. Kumashiro et al. (2026; Contribution 13), for example, apply the Michelangelo phenomenon to consumer relationships, proposing a model of product affirmation in which products, services, and brands may be able to function as symbolic relationship partners that affirm consumers’ desired traits, values, identities, and aspirations. Whether such symbolic affirmation produces developmental consequences comparable to affirmation from responsive human partners remains an open empirical question. Related questions concerning whether emerging conversational AI systems can perform some of the growth-supportive functions traditionally provided by human relationships are considered further in a later section.

Relationships can thus contribute to development proactively by encouraging exploration and goal pursuit, expanding the self, and affirming valued possible selves. As in adverse circumstances, however, the mere presence of a supportive other is unlikely to be sufficient; different interpersonal behaviors may facilitate different forms of growth, and their effectiveness may depend on characteristics of the individual, the relationship, and the goals being pursued. Moreover, adversity and opportunity frequently intersect: challenging goal pursuits can themselves generate stress, whereas periods of adversity may open possibilities for new identities, goals, relationships, or directions in life. The SOS and RC pathways therefore provide a useful analytical distinction, while recognizing that both may operate simultaneously within the same developmental experience.

## 5. Individual-Level Influences on Personal Growth

Although relationships and other socio-ecological systems play a central role in the personal growth process, individual differences in traits, belief systems, and abilities also influence personal growth processes, on their own or in conjunction with relational processes. As described already, various empirical contributions across this Special Issue illustrate a wide range of such influences, including attachment orientations, optimism and pessimism, humor styles, gender and social identities, and beliefs or orientations toward change. A couple of studies in this Special Issue also introduced new measures of individual characteristics that could be relevant to personal growth, such as the iterative mindset (Bobinet et al., 2025; Contribution 4) and the SOLACE Spectrum, a revised personality assessment intended for therapeutic and relational contexts that identifies six components (Rosenblad et al., 2025; Contribution 17). Such approaches reflect continued efforts to identify characteristics that may help explain why individuals differ in how they engage with opportunities and challenges, although further research is needed to establish how distinct these constructs are from existing measures and how well they predict personal growth.

Individual-level characteristics may be especially important in determining who benefits from experiences or activities intended to promote well-being or growth. The same activity need not benefit everyone and may, under some circumstances, be ineffective or even counterproductive. For example, Vannoy et al. (2026b; Contribution 24) identify different responses to a gratitude-letter intervention, including a subgroup for whom the intervention appeared to backfire, with decreases in positive affect and increases in negative affect. Similarly, a preregistered systematic map constructed by Gazder et al. (2025; Contribution 9) presents preliminary evidence that mindfulness, loving-kindness, and compassion-based interventions may be more beneficial for individuals higher in attachment anxiety than for those higher in attachment avoidance. These findings are consistent with broader positive-psychology intervention research suggesting that matching activities to individuals’ needs or preferences may improve some outcomes relative to assigning the same activity indiscriminately (e.g., Heintzelman et al., 2023).

Similar processes are likely to apply to research other than interventions. Characteristics of the individual may also result in individuals essentially creating an optimal or suboptimal environment in which to experience growth (Kumashiro et al., 2007). As discussed earlier, Gu et al. (2026; Contribution 11) present findings that greater attachment security was associated with a greater likelihood of experiencing the Michelangelo phenomenon. Similarly, differences associated with gender, identity, coping styles, and other person-level characteristics across contributions to this Special Issue suggest that apparently similar relational or life circumstances need not produce equivalent developmental outcomes. A key question is therefore not simply whether particular experiences, relationships, or interventions promote personal growth, but for whom they do so, under which circumstances, and through what processes.

Furthermore, temporal orientations in one’s identity may matter. Zhu et al. (2026; Contribution 25) present findings that future-self continuity, the extent to which individuals perceive their future self as connected to their current self, was associated with psychological well-being both directly and indirectly through meaning in life. Personal growth may also be aided by a meaningful look back at the past, but how one looks back makes a difference. Nostalgia, involving a sentimental connection to one’s personal past, was associated with personal growth, while declinism, or the general belief that society was better in the past, was associated with a stalled or reduced sense of growth (Feng et al., 2025; Contribution 8). In general, research on self-continuity distinguishes perceived connections between past and present, present and future, and the self across all three temporal perspectives, while also recognizing that some discontinuity can be adaptive when individuals seek to distance themselves from undesirable former selves (Sedikides et al., 2023). Personal growth may therefore involve a balance between sufficient continuity to provide coherence and meaning and sufficient openness to becoming different.

Taken together, these findings caution against treating personal growth as a uniform process. Individual differences in various characteristics may shape the opportunities people recognize and pursue, how they respond to adversity, the support they receive or can use effectively, and their responses to interventions intended to promote well-being or growth. They may also influence how change itself is interpreted: as continuity, development, loss, or movement toward a desired future self. To understand personal growth better, more research is needed to identify which processes are more likely to benefit different individuals and to examine potential mechanisms and moderators.

## 6. Future Research Directions

Recent developments in the personal growth literature, including the diverse selection in this Special Issue, highlight several priorities for future research. First, greater conceptual precision is needed regarding what researchers mean when they refer to personal growth. As discussed earlier, related theoretical traditions locate growth at different points in the developmental process: as an aspect of well-being, a motivation or intentional engagement in self-change, a belief about the possibility of change, an outcome following adversity, or a process supported by interpersonal and contextual conditions. While it may not be necessary to impose a single definition across these traditions, it would help to have a clearer picture of similarities and unique contributions across the disparate theories. This is especially important given broader evidence of increasing construct and measure proliferation within psychology (Anvari et al., 2025). This Editorial suggests that Feeney and Collins’s (2015) interpersonal model of thriving offers a useful organizing lens for understanding relational pathways to growth but acknowledges that their framework does not encompass all forms of personal growth processes. Future work should therefore prioritize conceptual mapping and tests of convergent, discriminant, and incremental validity rather than assuming that similarly named constructs are interchangeable or, conversely, introducing new constructs without establishing their relationship to existing ones.

Second, greater precision is needed in defining the circumstances in which growth occurs. Feeney and Collins’s (2015) distinction between thriving through adversity and thriving through life opportunities provides a useful analytical framework, but these domains may overlap frequently. For example, goal pursuit can itself be stressful, while adverse experiences may create opportunities to reconsider identities, relationships, priorities, and future goals. Similar boundary questions arise within PTG research. Although PTG developed as a theory of positive change arising through the struggle with trauma and highly challenging experiences, PTG concepts and measures have increasingly been applied across chronic stressors, indirect exposure to trauma, discrimination, and major life transitions. Contributions to this Special Issue illustrate this range, from self-reported traumatic experiences within the last five years, discrimination, parenthood, and vicarious exposure to others’ trauma. At the less severe end of this continuum, Cowden et al. (2025; Contribution 5) describe the development of the self-directed TRANSCEND workbook, intended as a scalable approach to addressing “everyday” suffering in nonclinical populations. Future research would benefit from more explicit assessment of the nature, severity, duration, and subjective disruption associated with growth-eliciting experiences, and from direct comparisons of growth processes across traumatic, stressful, and normative life circumstances.

Moreover, stronger empirical evidence is needed that measured personal growth reflects genuine change over time. This is especially pressing for PTG research, since the widely used Post-Traumatic Growth Inventory (PTGI; Tedeschi & Calhoun, 1996) relies on retrospective self-judgments of change that need not correspond to prospectively measured change (Frazier et al., 2009), prompting calls for longitudinal designs that distinguish perceived from demonstrated growth (Infurna & Jayawickreme, 2019). Contributions to this issue also raise questions of scoring: Taubman-Ben-Ari (2025; Contribution 21) presents findings that the 10-item PTGI-SF produced broadly similar associations to the full 21-item PTGI among first-time mothers, while Kruger (2026; Contribution 12) illustrates the value and potential psychometric shortcomings of examining PTGI domains separately. Future research should pair improved measurement with prospective, multi-timepoint designs and behavioral or observer indicators of growth.

Research should also place greater emphasis on determining for whom particular experiences or attempts to promote growth are beneficial and lead to better well-being. Several contributions to this Special Issue demonstrate the importance of considering individual differences, especially for interventions aimed at promoting personal growth and well-being (Gazder et al., 2025; Contribution 9; Vannoy et al., 2026b; Contribution 24). Other contributions show that attachment orientations, gender, identity, coping styles, temporal orientations, and moral identity may similarly qualify general growth-related processes. These findings converge with broader work emphasizing the person–environment fit more generally (Kandler et al., 2024). Future research should place more emphasis on examining the fit between various characteristics of the person and the type of personal growth processes that might yield positive outcomes. A related question concerns when personal growth actually contributes to subsequent well-being. Evidence that individuals have changed, or perceive themselves to have changed, should not itself be taken as evidence that such change improves well-being. Growth may be effortful or disruptive and may coexist with distress, making longitudinal research necessary to identify which forms of growth contribute to sustained well-being and who is more likely to benefit from such processes.

Furthermore, understanding personal growth requires widening the unit of analysis beyond the individual and the dyad. Ryff (2026; Contribution 18) emphasizes widening socioeconomic inequality as an obstacle to personal becoming, noting that opportunities to develop talents and capacities are not equally distributed. A socioecological perspective similarly emphasizes that psychological functioning is shaped by physical, interpersonal, economic, and political environments, rather than solely by individuals’ perceptions of those environments (Oishi, 2014). More research is needed, using multilevel and longitudinal designs, to examine how schools, workplaces, neighborhoods, relationship networks, and structural resources create or restrict opportunities for exploration and development, and how these contextual conditions interact with individual characteristics.

Relatedly, more attention is needed to assess how cultural differences and societal changes might affect research on personal growth. Contributions to this Special Issue draw on samples from several countries and cultural contexts, including from contrasting individualistic and collectivistic cultures such as the USA and China. Nevertheless, the presence of research across several countries and continents should not itself be taken as evidence that personal growth is conceptualized or experienced equivalently across cultures. Future research should directly compare cultural contexts, establish measurement equivalence where standardized measures are used, and investigate culturally valued forms of growth and culturally acceptable routes to pursuing them.

In particular, rapid societal changes, including changing expectations for education, employment, partnership, residential independence, and parenthood (e.g., Jager et al., 2022), may be altering both the opportunities for growth and the standards by which such development is judged. Declining rates of marriage and parenthood in many societies and the rise of singlehood as a meaningful identity (e.g., Kislev, 2024) may reflect a broader shift toward prioritizing autonomy, freedom of choice, and self-realization, although economic, structural, relational, and other factors are also important (e.g., Zaidi & Morgan, 2017). Whether changing life scripts create different opportunities or constraints for personal growth and who benefits the most remain unclear, and more research is needed on what changing social norms mean for research on personal growth. Future research should also consider whether the pursuit of personal growth can itself become counterproductive when individuals experience continual pressure to improve or construct an increasingly meaningful life. These questions are increasingly pressing as new technologies reshape who (or what) people turn to for support and companionship, as described next.

Finally, the rapid rise of AI technology and adoption may be changing how people experience and pursue personal growth, in a fast-moving landscape where research findings may become quickly obsolete. Three reviews in this Special Issue approach this from complementary perspectives: AI as a potential coach in educational and health contexts (Potel & Kumashiro, 2026; Contribution 16), and AI’s broader role in personal growth, well-being, and connection (Sharma et al., 2026; Contribution 19; Vannoy et al., 2026a; Contribution 23). Together, the review articles suggest that structured, purpose-built systems can offer short-term benefits, for certain tasks such as self-reflection and skill rehearsal, but warn of risks, especially in using more general-purpose large language models. Such AI systems are prone to inaccurate and sycophantic responding, while even structured AI applications and companions carry risks of emotional dependence, overreliance, reduced human connection, and skill erosion. In particular, such systems may provide feelings of responsive support while undermining the constructive challenge sometimes necessary for development. Other recent reviews emphasize risks of emotional dependence and displacement or devaluation of human relationships and argue that synthetic relationships may be most beneficial when complementing rather than replacing human connection (Smith et al., 2025; Ventura et al., 2026).

These developments make AI a useful test case for broader theories of personal growth rather than simply a new delivery mechanism for existing interventions. Future research might ask which growth-supportive functions artificial systems can actually provide. Can an AI system provide responsive support without becoming indiscriminately validating; encourage exploration in a secure-base-like manner; offer constructive challenge and accountability; or affirm a user’s own ideal self without imposing or reinforcing goals generated by the system? Can it function as a Source of Strength during adversity or as a Relational Catalyst for exploration and goal pursuit, and how would these functions differ from those provided by a reciprocal human relationship? Research on AI and growth therefore needs longer-term, ecologically valid studies comparing human, AI, and hybrid forms of support; attention to individual and contextual moderators; assessment of downstream effects on human relationships and capacities; and transparent reporting of the particular models and system features being studied.

Across these priorities, the central challenge is to move beyond demonstrating that people can report personal growth toward specifying what kind of growth has occurred, how it developed, for whom, under which interpersonal and socioecological conditions, and with what consequences over time. Personal growth is unlikely to be a uniform endpoint reached through a single pathway. It may instead be better understood as a family of related developmental processes and outcomes, the meaning and consequences of which depend on the person, the relationships and environments in which development occurs, and the cultural and historical context in which change is interpreted. Greater conceptual and methodological precision across these levels will be necessary to determine not simply whether people change, but when such change constitutes meaningful and sustainable personal growth.

## 7. Conclusions

In sum, the contributions to this Special Issue show that personal growth is not a single, uniform process, but can involve different forms of change arising through different pathways and life circumstances. This Editorial adopted an interpersonal framework to highlight the importance of relationships for personal growth. Growth may emerge through adversity or through engagement with opportunities, and its likelihood and consequences depend on individual characteristics, relationships, and broader social and cultural contexts. Experiences intended to promote growth are not invariably beneficial, nor should perceived growth automatically be equated with demonstrable change. Most importantly, the rapid pace of societal and technological changes may also reshape longstanding questions about what it means to experience personal growth and to live a meaningful life, and how such growth contributes to well-being.

## Data Availability

No new data were created or analyzed in this study. Data sharing is not applicable to this article.

## Note

1 Given the breadth of research relevant to personal growth, this editorial provides a selective rather than exhaustive review, focusing on theoretical traditions that are directly represented in, or provide useful frameworks for integrating, contributions to the Special Issue. Related literature on topics such as general goal pursuit, motivation, education, and work are therefore not reviewed comprehensively.
